# Hand hygiene and face mask wearing practices for COVID-19 prevention: a non-intrusive observation of patrons of community convenience shops in Accra, Ghana

**DOI:** 10.11604/pamj.2021.40.195.26195

**Published:** 2021-12-01

**Authors:** Donne Kofi Ameme, Magdalene Akos Odikro, Abraham Baidoo, Paul Dsane-Aidoo, Francis Sena Nuvey, Dikena Garmonyou Jackson, Abdul Gafaru Mohammed, Felicia Alemna, Emmanuel Kwame Tender, Georgia Naa Korkoi Ghartey, Oxygen Gershion Wullar, Leroy Maximore, Yaw Karikari Asamoah, Ernest Kenu

**Affiliations:** 1 Ghana Field Epidemiology and Laboratory Training Program, Accra, Ghana,; 2 School of Public Health, University of Ghana, Legon, Accra, Ghana,; 3 Swiss Tropical and Public Health Institute, University of Basel, Basel, Switzerland

**Keywords:** Adherence, COVID-19, face covering, handwashing, infection prevention

## Abstract

**Introduction:**

in the absence of a vaccine and definitive treatment, non-pharmacological measures of physical distancing, regular hand hygiene and wearing of face covering remain the mainstays of mitigating coronavirus disease 2019 (COVID-19). In Ghana, these measures are mandatory in public places and underpin COVID-19 infection prevention and control (IPC). We assessed adherence and appropriate performance of these measures among patrons of community convenience shops in selected hotspots.

**Methods:**

we conducted a non-intrusive observation of patrons of convenience shops in COVID-19 burden hotspots. We observed patrons as they entered and exited the shops and collected data on their gender, adherence and appropriate use of face covering and hand hygiene facilities. Data were analyzed descriptively and inferentially to determine factors associated with adherence to IPC guidelines.

**Results:**

of 800 patrons observed from eight community shops, 481 (60.1%) were males. Of these, 653 (81.6%) wore face covering and 98 (12.3%) performed hand hygiene; with 92 (11.5%) adhering to both measures. Patrons who wore face mask appropriately were 578; comprising 299 (92.3%) of patrons who wore face mask before entering the shops. Of 89 patrons who washed their hands, appropriate handwashing was recorded among nine (10.1%). Compared to inappropriate handwashing, appropriate handwashing was negatively associated with adherence to IPC guidelines [aOR=0.1 (95% CI=0.01-0.59)].

**Conclusion:**

adherence to and appropriate performance of IPC measures of handwashing and use of face covering in the selected shops was low. There is the need to complement availability of IPC measures with intensification of risk communication messaging targeted at ensuring their appropriate use.

## Introduction

Coronavirus disease 2019 (COVID-19) pandemic, caused by the novel severe acute respiratory syndrome coronavirus 2 (SARS-CoV-2) has affected all parts of the globe, with virtually no region unaffected [1]. While evidence on pharmacological treatments and vaccines is being generated to control the scourge of COVID-19, simple non-pharmacological measures such as physical distancing, eye protection, use of face mask and regular handwashing have been proven and accepted to be the mainstay in preventing person-to-person transmission [2-4]. Application of these measures at the community level, which have been strongly recommended [5,6] and incorporated in many national guidelines, will continue to be accepted adjuncts to other mitigating measures. High levels of adherence to this suite of preventive measures have been observed in some settings.

In Ghana, since the first two cases of COVID-19 were reported in March 2020 [7], several measures including ban on public gatherings, closure of schools and night clubs, closure of land, sea and air borders to human traffic, mandatory quarantine of travelers into the country and partial lockdown of selected cities considered as transmission hotspots were implemented. These measures were implemented together with enhanced surveillance activities such as active case finding, contact tracing and case management. While some of the restrictions have been gradually eased in accordance with expert criteria and recommendations on gradual reopening of societies [8,9], mandatory use of face mask, regular handwashing and physical distancing have dominated risk communication messaging as the potential game changers. These measures are expected to be in place in public places, commercial centers and all facilities accessible to the public regardless of whether public or privately owned [10]. All persons accessing such places as banking halls, offices and shops are expected to wear face covering and perform hand hygiene at hand hygiene stations available at the entrance of these places. In order to ensure adherence, audio-visual messages that reinforce handwashing and face mask use were disseminated in traditional and social media. Billboards on these measures have also been mounted along major streets in communities.

Although anecdotal evidence abounds on non-adherence of the general public to the laid down protocols for COVID-19 prevention in the country, there is hardly enough empirical evidence to support this claim. This study therefore sought to measure the adherence to and appropriate practice of infection prevention and control (IPC) measures of hand hygiene, face mask and face shield use in a well-controlled high traffic setting, as a basis for informing policy.

## Methods

**Study design:** a cross-sectional study was conducted across selected burden hotspots of COVID-19 in the Greater Accra Region.

**Study setting:** the study was conducted in the Greater Accra Region of Ghana which is considered the national epicenter of the COVID-19 pandemic. The region has a population of about 4 million [11] with 29 metropolitan, municipal and district assemblies. Accra, the capital city of the region, which is also the national capital is replete with several shops dotted in communities.

**Sample size and sampling:** using observed prevalence of face mask use of 11% [12,13], we estimated that a minimum of 777 patrons were required to be observed using the standard normal variate of 1.96, type I error of 5% and relative precision of 20%. We approximated the sample size to 800 to cater for questionnaires that may not be properly filled.

Selection of the districts and communities was on the basis of COVID-19 caseload at the time of the study. Based on the determined sample size and planned number of 100 patrons (50 entering and 50 exiting) to be allocated to each shop, eight districts were randomly selected for the study. Eight districts identified as burden hotspots for COVID-19 using existing COVID-19 caseload mappings were selected by simple random sampling from 10 burden hotspots. In each district, a community in the district capital with brisk commercial activities was selected. In each community, one convenience shop was purposively selected based on popularity and perceived high patronage by the community members. The selected shop should have a functional handwashing facilities including running water and soap as well and hand sanitizers as part of the directives required for operation during the COVID-19 pandemic. The shop should also not have a security personnel stationed at the entrance to ensure patrons adhere to the needed IPC measures. The sample size was uniformly distributed among the eight shops. Consecutive patrons entering the shop beginning with the first person were included in the study to be observed. Observations were done covertly by trained research assistants from July 22^nd^ to 25^th^, 2020.

**Data collection:** data was collected by health workers who were trained on the data collection procedure to ensure their observations were valid and inter-observer variabilities were minimized. At approximately 9 a.m. on the day of observation, observers went to the designated convenience shops in pairs and covertly observed patrons of the convenience shops from a distance, as they enter and exit the shops. One observer collected data on patrons entering the shop whilst the other collected data on those exiting the shop. Research assistants observed adult patrons for the use of face covering, the type of face covering worn, and whether face masks particularly were worn (covering both mouth and nostrils). They also observed whether the patrons observed hand hygiene (either by washing with soap and water or using hand sanitizers) and whether they went through the appropriate motions of performing hand hygiene which sought to cover all parts of the hands or any series of motions similar to World Health Organization (WHO) recommendations [14]. The observers recorded the gender of each patron based on the observed gross anatomical features and socially agreed upon characteristics. Data were recorded anonymously, using portable handheld device with open data kit (ODK) software. The observation was done from when the shops opened in the morning up to when the required 50 patrons were observed entering and exiting each shop. The data collection tool was pretested in a convenience shop in Ashaiman Municipality of the Greater Accra Region which shares similar characteristics as the other districts included in this study.

**Data management and analysis:** quality control checks were built into the data collection tool uploaded onto the ODK software. Data was exported to Microsoft Excel and then to Epi Info Version 7.2.1.0 for analysis. We performed descriptive analysis of the data by calculating frequencies and proportions of outcome variables: patrons who wore face covering, appropriately wore mask, performed hand hygiene and appropriately washed their hands. We categorized face mask use as appropriate if it was worn to cover both mouth and nostrils. Handwashing was deemed as appropriate if it was done in semblance of recommended WHO steps of handwashing or in a way that showed intention of the patron of covering all parts of the hands. We defined adherence to IPC measures as both the use of face covering and performance of hand hygiene. We calculated the frequency and proportion of patrons who adhered to both IPC measures (use of face mask and performance of hand hygiene) by gender. Patrons who wore face covering and also performed hand hygiene before entering the shops were considered to have adhered to IPC guidelines. We determined association between gender, appropriate wearing of face mask and appropriate handwashing as explanatory factors and adherence to IPC measures at 5% confidence level. Crude odds ratios (cOR), 95% confidence intervals and p-values were calculated. Variables that were significantly associated with adherence to COVID-19 IPC measures (with a p-value less than 0.05) at bivariable analysis were entered into a multivariable logistic regression analysis. Adjusted odds ratios (aOR) and 95% confidence intervals were calculated to determine factors independently associated with adherence to COVID-19 IPC measures.

**Ethical considerations:** the study was conducted as part of the COVID-19 response activities in Ghana. It was approved by the Ghana Health Service Ethics Review Committee (GHS-ERC 006/05/20). Given the likelihood of bias if patrons were aware that they were being observed, we did not seek prior consent from them.

## Results

**Description of the patrons observed:** a total of 800 patrons of convenience shops were observed of which 481 (60.1%) were males. Of 400 patrons observed entering the shops, 241 (60.3%) were males whilst 240 (60.0%) of the other 400 observed exiting were males. Data were obtained from all patrons (Table 1).

**Table 1:** gender distribution of patrons observed entering and exiting convenience shops by district, Greater Accra Region, 2020

District	Entry (n=400)		Exit (n=400)	
	Male	Female	Male	Female
Accra metropolitan area	34	16	26	24
Ayawaso West	37	13	37	13
Korle Klottey	45	5	42	8
Madina	16	34	17	33
Okaikoi North	31	19	31	19
Tema metropolitan area	14	36	23	27
Tema West	38	12	37	13
Weija Gbawe	26	24	27	23
Total	241	159	240	160

**Infection prevention and control practices of patrons observed:** of all patrons observed, 653 (81.6%) wore face covering and 98 (12.3%) performed hand hygiene whilst 92 (11.2%) adhered to both measures. Of patrons observed entering shops, 328 (82.0%) wore face covering whilst 325 (81.3%) of those observed exiting wore face covering. Patrons who performed hand hygiene either by using hand sanitizer or washing hands with soap and water before entering the shops were 91 (22.9%). Only 7 (1.8%) of the 400 patrons observed exiting the shops performed hand hygiene immediately after exiting. Of the patrons who wore face covering, 634 (79.3%) wore face mask either alone or together with face shield; with 572 (71.5%) of these number wearing the face masks to cover both mouth and nostrils (appropriately). Of all patrons who wore face covering, 215 (32.9%) of them wore only face shield and only five (0.8%) wore face shield in combination with face mask.

**Figure 1:**
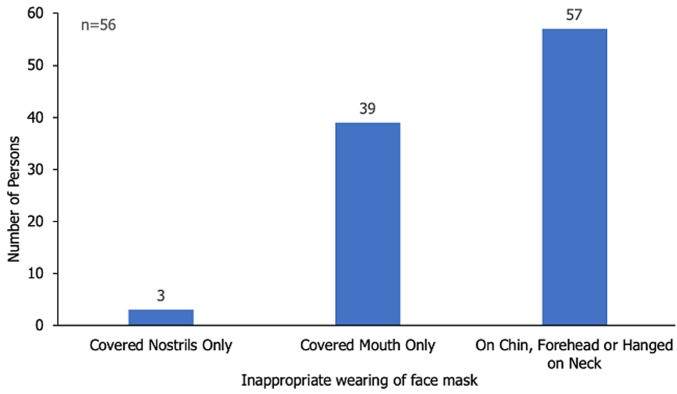
distribution of inappropriate ways of face mask donning among patrons of the convenience shops, Greater Accra Region, 2020

For those who did not wear the masks appropriately, 22 (39.2%) wore the masks to cover only their mouth whilst majority 32 (57.1%) had the masks on their chin or hanging on their neck (Figure 1). Patrons who washed their hands with soap and water as their form of hand hygiene were 89; comprising 84 (92.3%) and 5 (71.4%) of patrons who performed hand hygiene before entry and immediately after exit respectively. Only 9 (10.1%) of patients who washed their hands performed the chore appropriately (Table 2).

**Table 2:** infection prevention and control practices by patrons entering and exiting the convenience shops, Greater Accra Region, 2020

Infection prevention and control measure	Patron's activity		Total
	Entry n (%)	Exit n (%)	
**Face covering**			
Yes	328 (82.0)	325 (81.3)	653 (81.6)
No	72 (18.0)	75 (18.8)	147 (18.4)
Total	400 (100.0)	400 (100.0)	800 (100.0)
**Only face mask**			
Yes	320 (97.6)	309 (95.1)	629 (96.3)
No	8 (2.4)	16 (4.9)	24 (3.7)
Total	328 (100.0)	325 (100.0)	653 (100.0)
**Only face shield**			
Yes	200 (61.0)	15 (4.6)	215 (32.9)
No	128 (39.0)	310 (95.4)	438 (67.1)
Total	328 (100.0)	325 (100.0)	653 (100.0)
**Face shield and mask**			
Yes	4 (1.2)	1 (0.3)	5 (0.8)
No	324 (98.8)	324 (99.7)	648 (99.2)
Total	328 (100.0)	325 (100.0)	653 (100.0)
**Mask wearing**			
Appropriate	299 (92.3)	279 (90.0)	578 (91.2)
Inappropriate	25 (7.7)	31 (10.0)	56 (8.8)
Total	324 (100.0)	310 (100.0)	634 (100.0)
**Hand hygiene**			
Yes	91 (22.9)	7 (1.8)	98 (12.3)
No	307 (77.1)	393 (98.3)	700 (87.7)
Total	398 (100.0)	400 (100.0)	798 (100.0)
**Type of hand hygiene**			
Alcohol based hand gel	7 (7.7)	2 (28.6)	9 (9.2)
Handwashing	84 (92.3)	5 (71.4)	89 (90.8)
Total	91 (100.0)	7 (100.0)	98 (100.0)
**Hand washing**			
Appropriate	5 (6.0)	4 (80.0)	9 (10.1)
Inappropriate	79 (94.0)	1(20.0)	80 (89.9)
Total	84 (100.0)	5 (100.0)	89 (100.0)
**Touched faucet after handwashing**			
Yes	57 (67.9)	4 (80.0)	61 (68.5)
No	27 (32.1)	1 (20.0)	28 (31.5)
Total	84 (100.0)	5 (100.0)	89 (100.0)
**Both face covering and hand hygiene**			
Yes	86 (21.5)	6 (1.5)	92 (11.5)
No	314 (78.5)	394 (98.5)	708 (88.5)
Total	400 (100.0)	400 (100.0)	800 (100.0)

**Bivariable analysis:** of those who adhered to both IPC measures, 87 (94.6%) wore their masks appropriately and 6 (6.5%) washed their hands appropriately. Compared to females, being a male was positively associated with adherence to both IPC measures (hand hygiene and face covering use) at statistically significant level in bivariable analysis [cOR=2.3 (95% CI=1.39-3.78)] (Table 3).

**Multivariable analysis:** in the multivariable analysis, appropriate handwashing was negatively associated with adherence to COVID-19 preventive measures at statistically significant levels after adjusting for gender and appropriate mask wearing [aOR=0.1 (95% CI=0.01-0.59)] (Table 3).

**Table 3:** relationship between patron characteristics and adherence to IPC measures among patrons of convenience the shops, Greater Accra Region, 2020

Characteristic	Adherence to infection prevention and control measures		cOR (95% confidence interval)	p-value	aOR (95% confidence interval)	p-value
	Yes n (%)	No n (%)				
Gender			2.3 (1.39-3.78)	<0.001	1.02 (0.10-10.96)	0.98
Male (n=481)	70 (14.6)	411 (85.5)				
Female(n=319)	22 (6.9)	297 (93.1)				
Appropriate mask wearing			1.8 (0.70-4.66)	0.21		
Yes (n=578)	87 (15.1)	491 (85.0)				
No (n=56)	5 (8.9)	51 (91.1)				
Appropriate handwashing			0.1 (0.01-0.58)	0.03	0.1 (0.01-0.59)	0.01
Yes (n=6)	4 (66.7)	2 (33.3)				
No (n=83)	80 (96.4)	3 (3.6)				

cOR: crude odds ratio; aOR: adjusted odds ratio

## Discussion

The results of this study portray some level of disregard by the general public for COVID-19 preventive measures as has been observed elsewhere in the country [15]. The rising number of cases of COVID-19 in Ghana has been anecdotally blamed on the lack of adherence to IPC guidelines. Though the reasons for non-adherence were not explored in this study, a possibility could be the society´s disbelief of the existence and serious sequelae of COVID-19. This is likely because of the many conspiracy theories [16] that have flooded the media landscape and trickled into our communities. It is also likely that those who believe in the existence of the disease but were non adherent to the preventive measures perceived their risk of getting infected with the virus as low for one reason or the other. It would be worthwhile to launch a study into the reasons for non-adherence to preventive measures.

Concerning use of face covering, it is clear from the results that a large number of the patrons possessed and attempted donning the face mask. However, the fact that quite a number of them wore them inappropriately gives cause for concern especially because along the directive for mandatory use of face mask, came the procedure for donning and doffing [10]. It is likely that the accompanying guidelines on appropriate use of the face mask might not have gone down well. For this reason, even for those who seem to be adhering to these measures, they lack insight into the appropriate implementation. It appears that for some, possession of a face mask, hand sanitizer or face shield is erroneously considered enough protection regardless of how one uses them. This is evident by the display of inappropriate use of face mask in this study and elsewhere [17]. For those who were not wearing any face barrier, it is difficult to tell whether the additional cost of procuring face mask was what was deterring them from using one. Understandably, not everybody will be able to afford the face masks and face shields. However, in Accra where this study was conducted, face masks and face shields of all types are readily available for purchase in virtually all shops from street corner shops to large departmental shops. They are currently one of the commonly hawked items on the streets of Accra.

Though handwashing is a simple and primary preventive measure that most people are capable of doing independently [18], it is likely to be embraced as the new normal if the public understands and accepts the reasons for doing so. Same applies to other measures such as wearing of face mask and face shield. Of those who washed their hands before entering the shops, majority did not do that appropriately. Equally worrying is the large proportion of those who touched the faucet with their bare hands after washing their hands. These observations are not unexpected as educational messages on hand hygiene has emphasized the act rather than the art. Anecdotally, most people who wash their hands regularly at various hand hygiene stations at public places do so as a way of checking the box without going through the full motion to ensure all parts of their hands are covered. Even though majority of the patrons who washed their hands failed to go through the necessary motions involved in appropriate handwashing, the fact that about a quarter of them performed hand hygiene provides opportunity for improvement through education of the public on the appropriate way of performing hand hygiene. The level of adherence to hand hygiene, though left much to be desired, is not surprising as the patrons were not compelled to either use hand sanitizers or washed their hands before entering the shops. A much higher level of adherence would have been observed if the available hand hygiene stations were made prerequisites for entering the shops. This is because, elsewhere, provision of free access to hand hygiene stations in public health emergency settings and making their use mandatory has been shown to increase adherence [19].

WHO recommendation on hand hygiene for prevention transmission of COVID-19 requires that patrons perform hand hygiene not only before entering facilities accessible to the public but also after leaving [3]. However, hand hygiene at point of exit from such places as shops has been under-emphasized in risk communication messages reflecting in the fewer number of people observed to have either washed their hands or used hand sanitizers immediately after exiting the shops. In our study, we did not assess whether persons who performed hand hygiene while entering the shops were same persons who repeated the measure immediately after exit.

Though quite a large proportion of the patrons were non-adherent to the preventive measures, particularly performance of hand hygiene before entering shops, it is important to recognize that behavior change is a process and more likely to be sustained with reinforcement [20]. Of interest and surprising is the observation that being a male was positively associated with adherence to both hand hygiene and face covering use guidelines compared to females. The reasons for this are not known as females have been observed to more likely to wash their hands than males [21]. Also, a study done elsewhere found that males were less likely to wear face covering than females because males consider wearing them as shameful and a sign of weakness. This gender difference was however thought to have disappeared in settings where wearing of face covering was mandatory [22]. However, in our study, this difference still remained in spite of a ministerial directive on mandatory wearing of face covering in public places [10]. The association, which was significant in the bivariable analysis, was however, lost in both magnitude and significance in the multivariable analysis. The negative association of appropriate handwashing with adherence to COVID-19 IPC measures could be an indication of piecemeal adoption of preventive practices by the general population.

Regarding the use of alcohol-based hand rubs, it was not practicable to observe the appropriate application as the patrons who used them went into the shops immediately after dispensing them into the palm of their hands. Future studies should explore the use of alcohol-based hand rubs particularly those installed or offered at public places, to ensure the quantities dispensed are enough to perform a thorough hand hygiene and also observe the application. It is important to take cognizance of the limitations of this study while interpreting the findings. As a cross-sectional study, the findings were the practices prevailing at the time. These findings are not generalizable and could vary by geographic location and time. Also, there was a possibility of some observer biases which was minimized by standardizing how the observations were done with little observer subjectivity. In addition, it was possible that the gender of the patrons could have been misclassified considering the fact that this determination was based on a brief observation. This is however, almost certainly very minimal as toddlers were excluded from the study.

The possibility of Hawthorne effect is minimized by not seeking consent from the patrons and also by using portable handheld devices for recording our observation instead of papers and pen. However, it is likely that just by the mere presence of observers in the vicinity of the shops, some patrons could be forced to put up the most acceptable behavior. Even though differences in adherence to face mask and face shield use, as well as handwashing practices were noticed between the various community shops, we refrained from drawing conclusions from that as our study was powered to use pooled results from all the shops. A larger multicenter study powered to detect such differences is worth considering. Despite these, our study provides valuable information which can be used for guiding risk communication strategies and informing policy.

## Conclusion

Proportion of patrons who adhered to and appropriately performed IPC guidelines of handwashing and use of face covering in the selected convenience shops was low. The results show that possession of and access to the necessary IPC materials for preventing COVID-19 is not synonymous to practice of appropriate IPC measures. In situations where specific measures were performed, they were largely inappropriately performed calling for renewed considerations for risk communication messaging for COVID-19 prevention to the general public. Understanding the reasons for non-adherence and incorrect practice of these measures would be a valuable add-on. Existing policy on mandatory face mask use and regular handwashing should be accompanied with communication campaign emphasizing why and how these should be done.

### What is known about this topic

- Wearing of face covering in public places where physical distancing is not possible prevents COVID-19 transmission;
- Performing hand hygiene by either handwashing with soap under running water or use of alcohol-based hand sanitizer prevents transmission of COVID-19.

### What this study adds

- Adherence to directives of wearing face mask in public places and performance of hand hygiene is not optimum;
- Even in instances where the public seems to adhere, many persons either do not wear face coverings appropriately or perform hand hygiene appropriately.
